# Supplementary material for: Molecular mechanism of antibody neutralization of coxsackievirus A16
Source: Nat Commun. 2022 Dec 21;13:7854. doi: 10.1038/s41467-022-35575-w (PMC9769477; doi:10.1038/s41467-022-35575-w)
Supplement: Supplementary file 1 — Supplementary information [file 41467_2022_35575_MOESM1_ESM.pdf]

# Supplementary Information for:

## Molecular mechanism of antibody neutralization of coxsackievirus A16

**This Supplementary Information PDF includes:**

**Figures S1 –S9**

**Tables S1 –S6**

**a**

| MAb | Heavy chain variable region | | | | Light chain variable region | | |
|---|---|---|---|---|---|---|---|
| | V | D | J | Identity (%) | V | J | Identity (%) |
| 8C4 | 1-82*01 | 1-1*01 | 2*01 | 95.6 | 13-85*01 | 2*01 | 99.3 |
| 9B5 | 1-22*01 | 2-4*01 | 4*01 | 92.2 | 12-46*01 | 4*01 | 96.8 |
| 9G1 | 14-4*02 | 2-4*01 | 1*01 | 100 | 8-24*01 | 1*01 | 94.3 |

**b**

```
                             20        CDR1             40
8C4-VH  QVQLQQSGPELVKPGASVKISCKASGYAFSTSWMNWVIQRPGQGLEWIGR 50
9B5-VH  E..................M...T...T.TENT.H..R.SH.KS.....G 50

         CDR2     60                   80                  100
8C4-VH  IYPGDGDTNYNGKFKGKATLTADKSSSTAYMQLSSLTSVDSAVYFCARRD 100
9B5-VH  ...KND..K..Q.........V.......C.E.R....E.....Y...G. 100

          CDR3           120
8C4-VH  Y-GYF---DYWGQGTTLTVSS 117
9B5-VH  .EN..YAM.......SV.... 121
```

**c**

```
                            20        CDR1            40
8C4-VL  DIQMTQSSSYLSVSLGGRVTITCKASDHINNWLAWYQQKPGNAPRLLISG 50
9B5-VL  .......PAS....V.ET.....R..EN.YSN.......Q.KS.Q..VYA 50

        CDR2        60                   80       CDR3     100
8C4-VL  ATSLETGVPSRFSGSGSGKDYTLSITSLQTEDVATYYCQQYWNSPYTFGG 100
9B5-VL  ..N.AD............TQ.S.K.N...S..FG......F.DT.F...S 100

8C4-VL  GTKLEIK 107
9B5-VL  ....A.. 107
```

**Supplementary Figure 1.** Sequence analysis of the anti-CVA16 MAbs. **(a)** The closest mouse germline genes were identified using NCBI IgBLAST. The percentage identity of nucleotide sequences of variable regions was also shown. **(b-c)** Amino acid sequences of heavy-chain variable regions (VH) (**b**) and light-chain variable regions (VL) (**c**) of the 8C4 and 9B5 antibodies. Dots represent residues identical to those of antibody 8C4, and red dashes are gaps. Locations of complementarity determining regions (CDR) were obtained from NCBI IgBLAST.

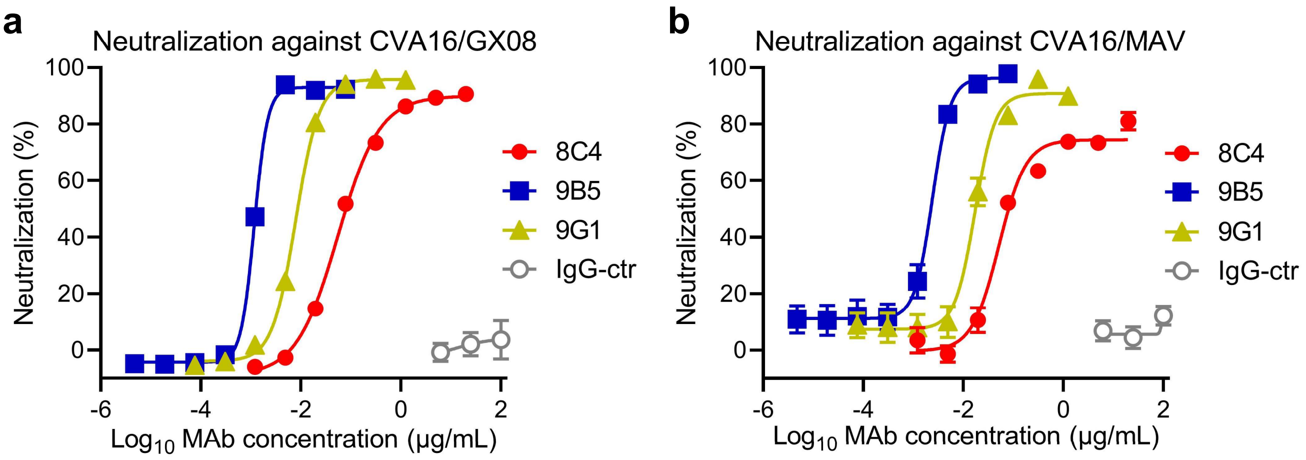


**Supplementary Figure 2.** Neutralization of the MAbs against CVA16 strains GX08 **(a)** and MAV (**b**) was measured by the cell viability assay. Related to Figure 1A. IgG-ctr, anti-SARS-CoV-2 MAb 2H2. Data are mean ± SEM of five replicate wells in 96-well cell culture plates.

**a**

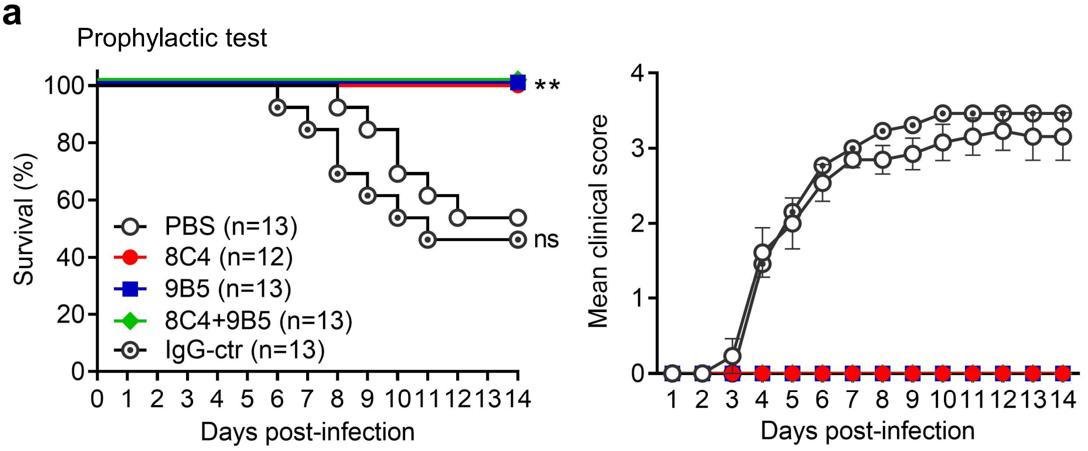


**b**

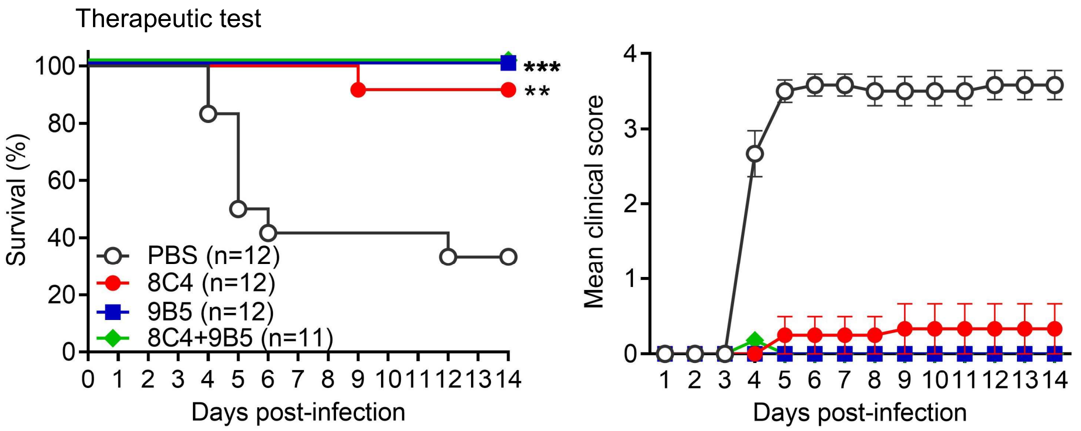


**c**

| Survival rate | First experiment | | Second experiment | |
|---|---|---|---|---|
| | PBS group | IgG-ctr group | PBS group | IgG-ctr group |
| Prophylactic test | 46.2% | 46.2% | 53.8% | 46.2% |
| Therapeutic test | 30.8% | Not tested | 33.3% | Not tested |

**Supplementary Figure 3.** In vivo prophylactic efficacy (**a**) and therapeutic efficacy (**b**) of MAbs 8C4, 9B5 and the 8C4+9B5 cocktail against CVA16 infection in mice. Results of two independent experiments are shown in Figure 2 and Supplementary Figure 3, respectively. Clinical scores were graded as follows: 0, healthy; 1, reduced mobility; 2, limb weakness; 3, limb paralysis; 4, death. The number of mice in each group were indicated in the bracket. Survival rates of MAb-treated mice were compared with the mice in the PBS-treated group. Statistical significance was determined by Log-rank (Mantel-Cox) test. ns, no significant difference ($p \geq 0.05$); **, $p < 0.01$; ***, $p < 0.001$. For panel **a**, p value between the PBS group and the 8C4 group is 0.0082; p value between the PBS group and the 9B5 group is 0.0060; p value between the PBS group and the 8C4+9B5 group is 0.0060. For panel **b**, p value between the PBS group and the 8C4 group is 0.0025; p value between the PBS group and the 9B5 group is 0.0006; p value between the PBS group and the 8C4+9B5 group is 0.0010. All error bars represent SEM. (**c**) The survival rates of mice in PBS and IgG-ctr groups.

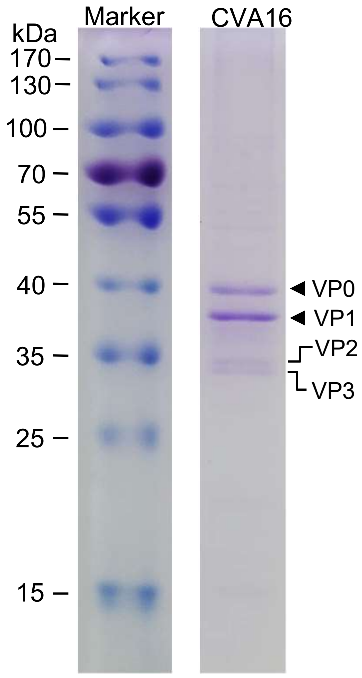


**Supplementary Figure 4.** SDS-PAGE analysis of purified CVA16. Note that the content of VP0 protein was far more than that of VP2 protein. Only one batch of CVA16 viral particles was prepared and purified.

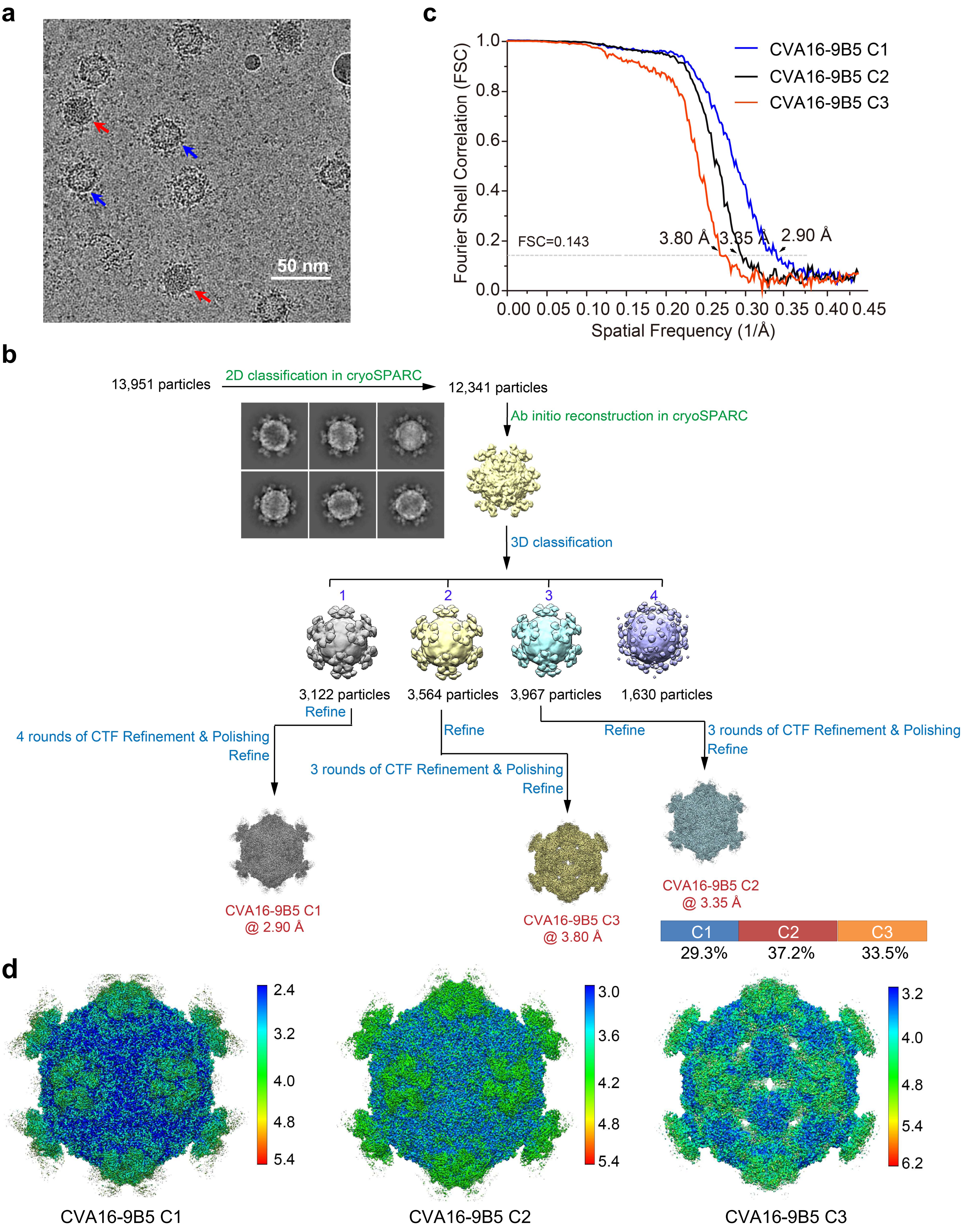


**Supplementary Figure 5.** Cryo-EM structural analysis of the CVA16–9B5 complex. (**a**) A representative cryo-EM image of the CVA16–9B5 complex. Red and blue arrows indicate full and empty CVA16 viral particles engaged by 9B5 Fabs, respectively. Two independent experiments were performed, with similar results. (**b**) Data processing procedure for the CVA16–9B5 dataset. Population distribution of CVA16–9B5 C1 to C3 is also shown. (**c**) Resolution estimation of the CVA16–9B5 C1 to C3 maps according to the gold-standard FSC criterion of 0.143. (**d**) Local resolution evaluations for the CVA16–9B5 C1 to C3 maps.

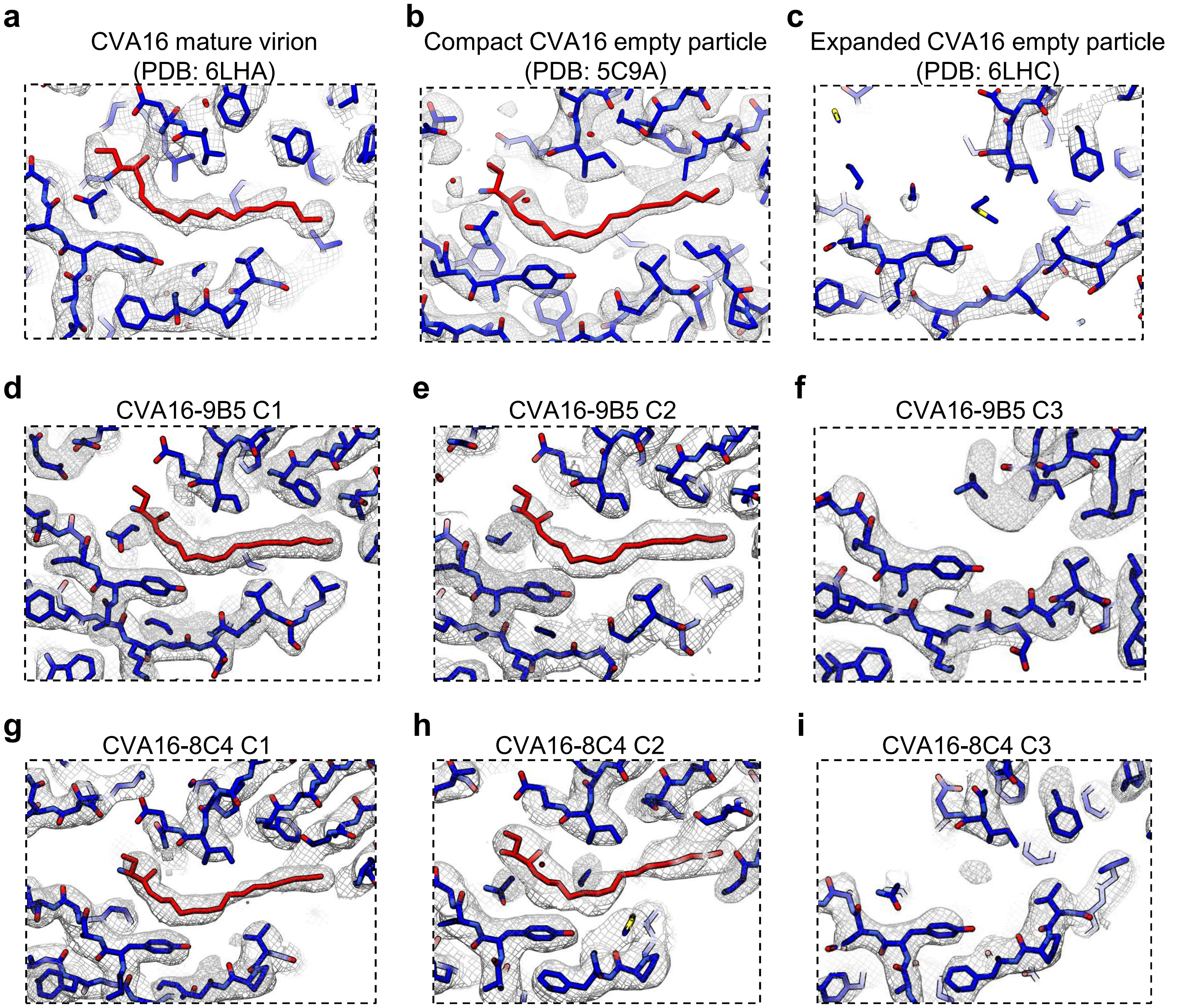


**Supplementary Figure 6.** Representative density maps of the VP1 pocket region in the CVA16 mature virion (**a**), compact empty particle (**b**), expanded empty particle (**c**), CVA16–9B5 C1 to C3 (**d-f**), and CVA16–8C4 C1 to C3 (**g-i**). Density is shown in gray mesh. The atomic models for VP1 and pocket factor are shown as blue and red sticks, respectively.

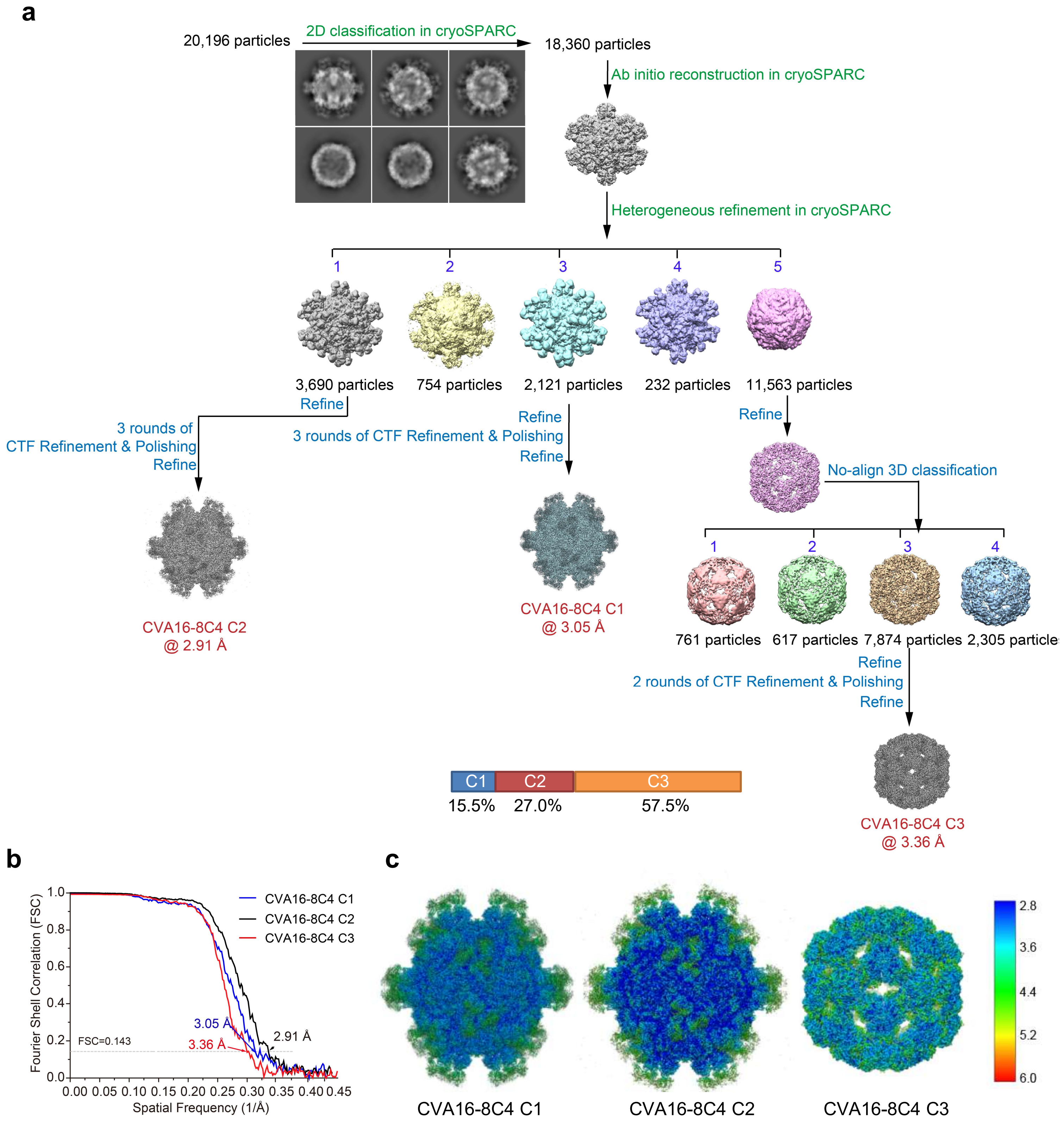


**Supplementary Figure 7.** Cryo-EM structural analysis of the CVA16–8C4 complex. (**a**) Data processing procedure for the CVA16–8C4 dataset. Population distribution of CVA16–8C4 C1 to C3 is also shown. (**b**) Resolution estimation of the CVA16–8C4 C1 to C3 maps according to the gold-standard FSC criterion of 0.143. (**c**) Local resolution evaluations for the CVA16–8C4 C1 to C3 maps.

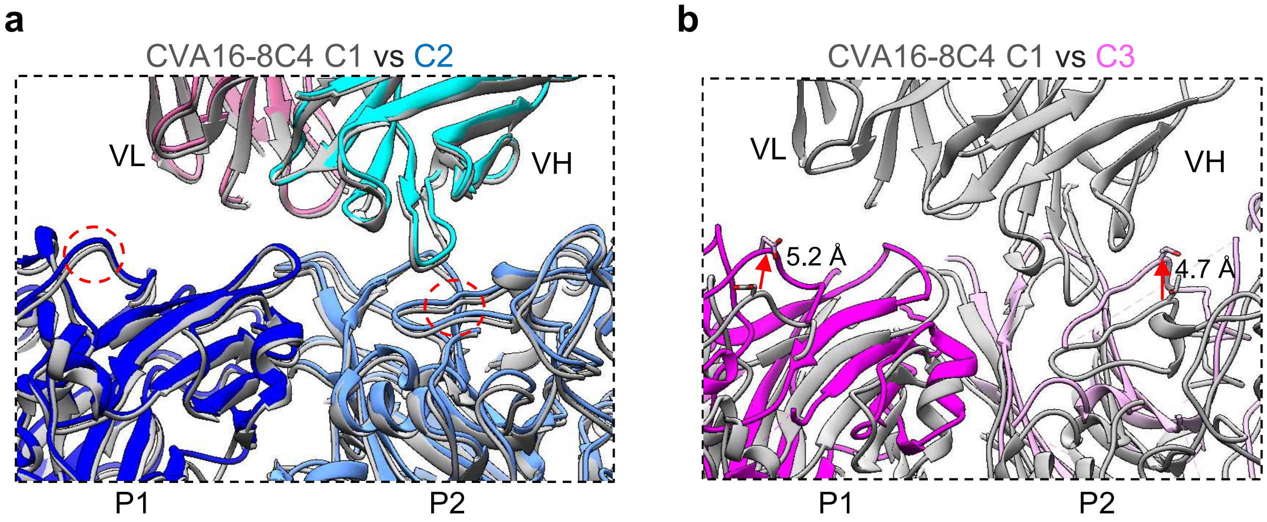


**Supplementary Figure 8.** Structural basis of antibody 8C4 binding to CVA16 capsid in the C2 structure but not in C3. (**a**) Superposition of the CVA16-8C4 C1 (gray) and C2 (color) structures. The red circles indicate slight movements of the capsid in the C2 structure relative to the C1 structure. (**b**) Docking of the C3 structure (magenta) into the C1 structure (gray). The red arrow represents the movement direction of the capsid in the C3 structure relative to the C1 structure.

**a**

| MAb | Antibody-bound CVA16 particle type | Epitope | Overlap with | Block | Neutralization mechanism |
|---|---|---|---|---|---|
| 9B5 | Compact mature virion<br>Compact empty particle<br>Expanded empty particle | VP1 BC, EF, HI loops | HSPG binding site | HSPG and SCARB2 binding | Mainly by blocking viral attachment; also by blocking SCARB2 binding |
| 8C4 | Compact mature virion<br>Compact empty particle | VP3 BC, HI loops;<br>VP2' βB, βC;<br>VP2' BC, EF, HI loops | SCARB2 binding site | SCARB2 binding | Mainly at an early post-attachment step (e.g. blocking SCARB2 binding) |

**b**

Anti-CVA16 MAbs
- Group 1 { 9B5, 18A7 } (5-fold axis)
- Group 2: NA9D7 (2-fold axis)
- Group 3 { 8C4, 14B10 } (3-fold axis)

**d**

| MAb | Contact area ($Å^2$) per Fab | Number of binding sites per viral particle |
|---|---|---|
| 9B5 | 536.4 | 60 |
| 18A7 | ~1184 | 12 |
| NA9D7 | ~761 | 60 |
| 8C4 | 904.3 | 60 |
| 14B10 | ~1200 | 60 |

**c**

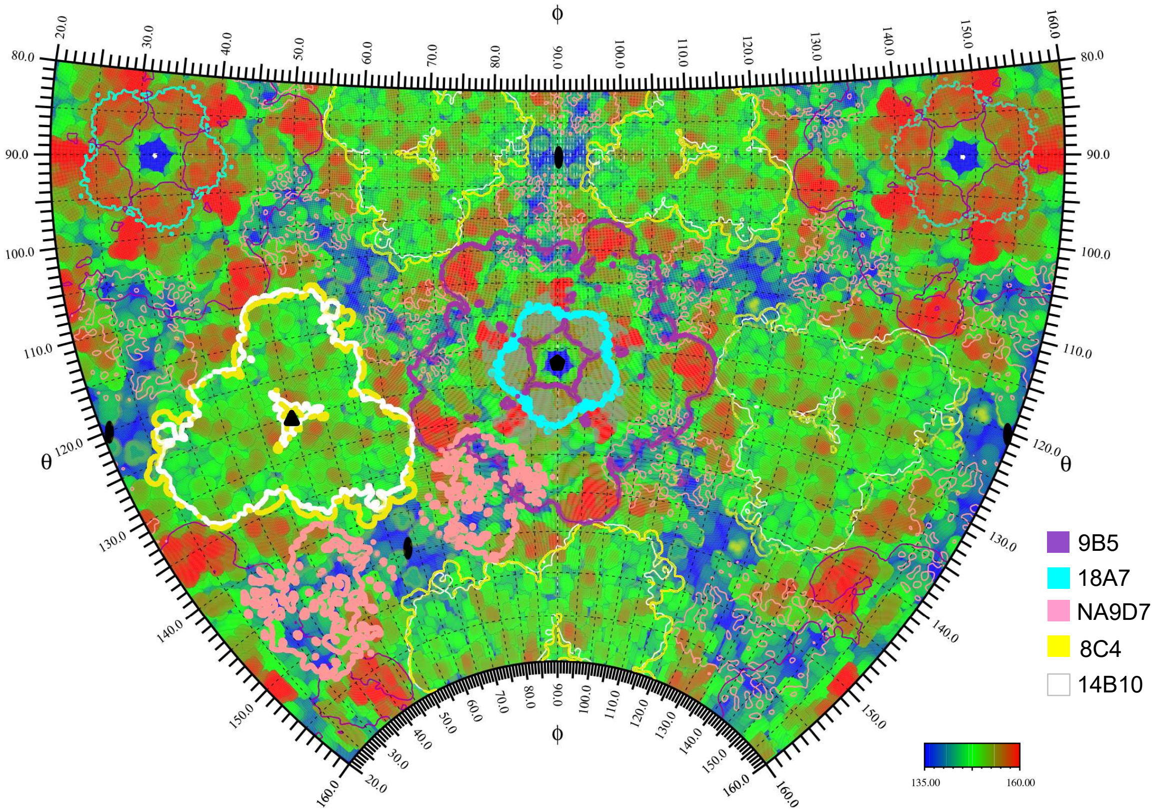


**e**

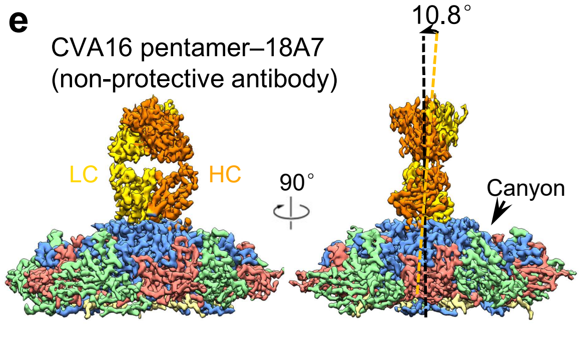


**f**

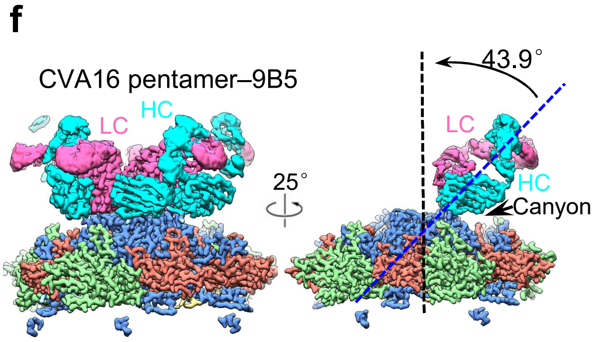


**Supplementary Figure 9.** Summary of information regarding the reported CVA16 MAbs with resolved structures. (**a**) Summary of epitopes and neutralization mechanisms of MAbs 8C4 and 9B5. (**b**) Grouping of anti-CVA16 MAbs. Antibody binding regions are shown in brackets. (**c**) Roadmap showing the footprints of the Fabs on the CVA16 virion surface. The 9B5, 18A7, NA9D7, 8C4, and 14B10 Fabs are indicated by purple, cyan, pink, yellow, and white contour lines, respectively. Surface area of CVA16 viral capsid covered by each Fab was also shown. (**d**) The surface area of contact between each Fab and CVA16 viral capsid.

(**e**) Cryo-EM densities of 5-fold vertex of CVA16 mature virion and a single bound 18A7 Fab. The angle between the long axis (gold dashed line) of the 18A7 Fab and the 5-fold axis (black dashed line) of viral capsid is shown. VP1, VP2, VP3, and VP4 are colored in cornflower blue, light green, salmon, and khaki, respectively. HC and LC of 18A7 are colored in orange and gold, respectively. (**f**) Cryo-EM densities of 5-fold vertex of CVA16 and five bound 9B5 Fabs. On the right panel, the densities of four 9B5 Fabs are removed for clarity. The angle between the long axis (blue dashed line) of the 9B5 Fab and the 5-fold axis (black dashed line) of viral capsid is shown.

**Supplementary Table 1.** A summary of all the CVA16 strains used in this study.

| CVA16 strains | Genotype | Genbank ID |
|---|---|---|
| CVA16/SZ05 [a] | B1b | EU262658 |
| CVA16/GX08 | B1b | KC342228 |
| CVA16/MAV | B1a | KC695830 |

[a] used as the antigen for mouse immunization.

**Supplementary Table 2.** Cryo-EM data collection and refinement statistics.

| | CVA16–9B5 | | | CVA16–8C4 | | |
|---|---|---|---|---|---|---|
| **Data collection and processing** | | | | | | |
| Magnification | 64,000 | | | 64,000 | | |
| Voltage (kV) | 300 | | | 300 | | |
| Detector | K3 | | | K3 | | |
| Electron exposure ($e^{-}/Å^2$) | 38 | | | 50 | | |
| Defocus range (μm) | -0.8 to -2.5 | | | -0.8 to 2.5 | | |
| Pixel size (Å) | 1.1 | | | 1.1 | | |
| Symmetry imposed | I | | | I | | |
| Structures | C1 | C2 | C3 | C1 | C2 | C3 |
| Initial particle images (no.) | 13,951 | 13,951 | 13,951 | 20,196 | 20,196 | 20,196 |
| Final particle images (no.) | 3,122 | 3.967 | 3,564 | 2,121 | 3,690 | 7,874 |
| Map resolution (Å) | 2.90 | 3.35 | 3.80 | 3.05 | 2.91 | 3.36 |
| FSC threshold | | | | | | |
| Map resolution range (Å) | 2.4-4.0 | 3.0-4.2 | 3.2-4.8 | 2.6-4.4 | 2.6-4.4 | 2.8-4.4 |
| **Refinement** | | | | | | |
| Initial model used (PDB code) | 5C4W | 5C9A | 6LHC | 5C4W | 5C9A | 6LHC |
| Model resolution (Å) | 3.0 | 3.2 | 3.7 | 3.5 | 3.1 | 3.4 |
| FSC threshold | | | | | | |
| Map sharpening *B* factor ($Å^2$) | -60.7 | -73.6 | -88.4 | -78.3 | -69.3 | -98.0 |
| Model composition | | | | | | |
| Non-hydrogen atoms | 9,720 | 9,091 | 7,822 | 9,720 | 9,049 | 4,477 |
| Protein residues | 1,258 | 1,174 | 1,010 | 1,258 | 1167 | 572 |
| Ligands | 1 | 1 | | 1 | 1 | |
| *B* factors ($Å^2$) | | | | | | |
| Protein | 73.9 | 85.0 | 108.72 | 55.40 | 67.80 | 74.23 |
| Ligand | 31.2 | 42.3 | | 33.81 | 33.81 | |
| R.m.s. deviations | | | | | | |
| Bond lengths (Å) | 0.007 | 0.008 | 0.006 | 0.011 | 0.010 | 0.003 |
| Bond angles (°) | 0.919 | 0.976 | 0.952 | 0.761 | 0.755 | 0.709 |
| **Validation** | | | | | | |
| MolProbity score | 1.62 | 1.72 | 1.65 | 1.54 | 1.40 | 1.40 |
| Clashscore | 4.70 | 5.98 | 6.08 | 4.77 | 3.76 | 3.70 |
| Poor rotamers (%) | 0.65 | 0.59 | 0.34 | 0.14 | 0.20 | 0.00 |
| Ramachandran plot | | | | | | |
| Favored (%) | 94.45 | 94.14 | 95.43 | 95.78 | 96.45 | 96.38 |
| Allowed (%) | 5.47 | 5.26 | 4.57 | 4.22 | 3.55 | 3.62 |
| Disallowed (%) | 0.08 | 0.60 | 0.00 | 0.00 | 0.00 | 0.00 |

**Supplementary Table 3.** RMSD values between the capsid protomers of different CVA16 structures.

| RMSD (Å) | CVA16–9B5 C1 | CVA16–9B5 C2 | CVA16–9B5 C3 | CVA16–8C4 C1 | CVA16–8C4 C2 | CVA16–8C4 C3 |
|---|---|---|---|---|---|---|
| Mature CVA16 virion (PDB: 5C4W) | **1.051** | 1.204 | 2.310 | **1.098** | 1.212 | 2.297 |
| Compact CVA16 empty particle (PDB: 5C9A) | 1.212 | **1.162** | 2.313 | 1.194 | **1.178** | 2.264 |
| Expanded CVA16 empty particle (PDB: 6LHC) | 2.314 | 2.428 | **1.240** | 2.294 | 2.258 | **0.937** |

**Supplementary Table 4.** Interaction interface analysis of the CVA16–9B5 C1 structure (upper table) and conservation analysis of the contacting residues (lower table).

| CVA16 | | Distance (Å) | 9B5 | | Interaction |
|---|---|---|---|---|---|
| Residue | Location | | Residue | Location | |
| G99 [N] | VP1 BC loop | 3.02 | N103 [OD1] | HCDR3 | H-bond |
| G99 [O] | VP1 BC loop | 3.07 | Y104 [N] | HCDR3 | H-bond |
| D104 [OD2] | VP1 BC loop | 2.73 | K59 [NZ] | HFR3 | H-bond, salt bridge |
| D104 [OD1] | VP1 BC loop | 3.62 | K59 [NZ] | HFR3 | Salt bridge |
| R166 [NH2] | VP1 EF loop | 2.90 | D93 [OD2] | LCDR3 | H-bond, salt bridge |
| R166 [NH1] | VP1 EF loop | 3.96 | D93 [OD1] | LCDR3 | Salt bridge |
| K242 [NZ] | VP1 HI loop | 2.84 | W92 [O] | LCDR3 | H-bond |

| 9B5-binding residues | | Number of sequences | Conservation |
|---|---|---|---|
| VP1 (1127 sequences) [a] | G99 | 1122 | 99.6% |
| | D104 | 1105 | 98.0% |
| | R166 | 1127 | 100% |
| | K242 | 1126 | 99.9% |

[a] Complete or near-complete CVA16 capsid protein sequences were obtained from NCBI database and used for conservation analysis.

**Supplementary Table 5.** Surface area of CVA16 viral capsid covered by MAb 9B5 and 8C4 (in C1 structure) determined using PISA.

| MAb | Interface area (Å$^2$) | |
|---|---|---|
| | Total | Contribution |
| 9B5-VH | 275.0 | 51.3% |
| 9B5-VL | 261.4 | 48.7% |
| 9B5 | 536.4 | 100.0% |
| 8C4-VH | 508.1 | 56.2% |
| 8C4-VL | 396.2 | 43.8% |
| 8C4 | 904.3 | 100.0% |

**Supplementary Table 6.** Interaction interface analysis of the CVA16–8C4 C1 structure (upper table) and C2 structure (middle table) and conservation analysis of the contacting residues (lower table).

| C1 structure | CVA16 | | Distance (Å) | 8C4 | | Interaction |
|---|---|---|---|---|---|---|
| | Residue | Location | | Residue | Location | |
| Protomer 1 | S77 [O] | VP3 BC loop | 3.17 | R99 [NH2] | HCDR3 | H-bond |
| | S77 [O] | VP3 BC loop | 3.32 | R99 [NH1] | HCDR3 | H-bond |
| | T210 [OG1] | VP3 HI loop | 3.07 | N30 [OD1] | LCDR1 | H-bond |
| Protomer 2 | S230 [OG] | VP2 HI loop | 3.28 | W92 [O] | LCDR3 | H-bond |
| | G227 [O] | VP2 HI loop | 3.07 | W92 [NE1] | LCDR3 | H-bond |
| | A228 [O] | VP2 HI loop | 3.43 | W92 [NE1] | LCDR3 | H-bond |
| | K69 [NZ] | VP2 βB | 2.73 | D55 [OD2] | HCDR2 | H-bond, salt bridge |
| | W78 [NE1] [a] | VP2 βC | 3.55 | D57 [OD2] | HCDR2 | H-bond |
| | V159 [N] | VP2 EF loop | 3.49 | D55 [O] | HCDR2 | H-bond |
| | D74 [OD1] | VP2 BC loop | 3.73 | Y60 [N] | HFR3 | H-bond |
| | D74 [OD1] | VP2 BC loop | 3.60 | N59 [ND2] | HFR3 | H-bond |
| | D74 [OD2] | VP2 BC loop | 3.72 | N59 [ND2] | HFR3 | H-bond |
| | E231 [OE1] [a] | VP2 HI loop | 3.22 | R99 [NH2] | HCDR3 | H-bond, salt bridge |

| C2 structure | CVA16 | | Distance (Å) | 8C4 | | Interaction |
|---|---|---|---|---|---|---|
| | Residue | Location | | Residue | Location | |
| Protomer 1 | S77 [O] | VP3 BC loop | 3.61 | R99 [NH2] | HCDR3 | H-bond |
| | S77 [O] | VP3 BC loop | 3.51 | R99 [NH1] | HCDR3 | H-bond |
| | T210 [OG1] | VP3 HI loop | 2.83 | N30 [ND2] | LCDR1 | H-bond |
| Protomer 2 | S230 [OG] | VP2 HI loop | 2.82 | W92 [O] | LCDR3 | H-bond |
| | S230 [N] | VP2 HI loop | 3.55 | N93 [OD1] | LCDR3 | H-bond |
| | G227 [O] | VP2 HI loop | 3.28 | W92 [NE1] | LCDR3 | H-bond |
| | A228 [O] | VP2 HI loop | 3.49 | W92 [NE1] | LCDR3 | H-bond |
| | K69 [NZ] | VP2 βB | 2.70 | D55 [OD2] | HCDR2 | H-bond, salt bridge |
| | V159 [N] | VP2 EF loop | 3.58 | D55 [O] | HCDR2 | H-bond |
| | D74 [OD1] | VP2 BC loop | 2.64 | Y60 [N] | HFR3 | H-bond |
| | D74 [OD1] | VP2 BC loop | 2.87 | N59 [ND2] | HFR3 | H-bond |

| 8C4-binding residues | | Number of sequences | Conservation |
|---|---|---|---|
| VP2 (399 sequences) [b] | K69 [c] | 398 | 99.7% |
| | D74 | 397 | 99.5% |
| | W78 | 399 | 100% |
| | V159 [c] | 397 | 99.5% |
| | G227 | 399 | 100% |
| | A228 | 399 | 100% |
| | S230 | 399 | 100% |
| | E231 | 399 | 100% |
| VP3 (191 sequences) [b] | S77 | 191 | 100% |
| | T210 | 191 | 100% |

[a] The interaction exists in the C1 structure, but not in the C2 structure.

[b] Complete or near-complete CVA16 capsid protein sequences were obtained from NCBI database and used for conservation analysis.

[c] The residues mutated in the neutralization escape mutants.
